# Pathogenicity and peramivir efficacy in immunocompromised murine models of influenza B virus infection

**DOI:** 10.1038/s41598-017-07433-z

**Published:** 2017-08-04

**Authors:** Philippe Noriel Q. Pascua, Heba H. Mostafa, Bindumadhav M. Marathe, Peter Vogel, Charles J. Russell, Richard J. Webby, Elena A. Govorkova

**Affiliations:** 1 Department of Infectious Diseases, St. Jude Children’s Research Hospital, Memphis, Tennessee USA; 2 Veterinary Pathology Core, St. Jude Children’s Research Hospital, Memphis, Tennessee USA

## Abstract

Influenza B viruses are important human pathogens that remain inadequately studied, largely because available animal models are poorly defined. Here, we developed an immunocompromised murine models for influenza B virus infection, which we subsequently used to study pathogenicity and to examine antiviral efficacy of the neuraminidase inhibitor peramivir. We studied three influenza B viruses that represent both the Yamagata (B/Massachusetts/2/2012 and B/Phuket/3073/2013) and Victoria (B/Brisbane/60/2008, BR/08) lineages. BR/08 was the most pathogenic in genetically modified immunocompromised mice [BALB *scid* and non-obese diabetic (NOD) *scid* strains] causing lethal infection without prior adaptation. The immunocompromised mice demonstrated prolonged virus shedding with modest induction of immune responses compared to BALB/c. Rather than severe virus burden, BR/08 virus-associated disease severity correlated with extensive virus spread and severe pulmonary pathology, stronger and persistent natural killer cell responses, and the extended induction of pro-inflammatory cytokines and chemokines. In contrast to a single-dose treatment (75 mg/kg/day), repeated doses of peramivir rescued BALB *scid* mice from lethal challenge with BR/08, but did not result in complete virus clearance. In summary, we have established immunocompromised murine models for influenza B virus infection that will facilitate evaluations of the efficacy of currently available and investigational anti-influenza drugs.

## Introduction

Immunocompromised individuals are highly susceptible to influenza virus infections, having prolonged virus shedding, extended hospitalizations, and often severe complications^1–5^. The two major options for controlling influenza infection, namely vaccines and antiviral treatment, have limitations in immunocompromised patients. These patients respond poorly to influenza vaccination, emphasizing the leading role of neuraminidase inhibitors (NAIs), the only class of antiviral drugs recommended for prophylaxis against and treatment of influenza^1^. Prolonged viral shedding increases the risk of drug-resistant variants emerging^6–10^, and the development of resistance has been documented in immunocompromised patients treated with NAIs^5, 7, 9, 11–15^.

Influenza B viruses are important seasonal human respiratory pathogens that co-circulate alongside influenza A viruses worldwide. However, they remain largely understudied, mainly because their prevalence tends to cycle by season and they are only infrequently dominant^16–18^. In contrast to influenza A viruses, which infect a broad spectrum of animals, influenza B viruses have a limited host range and are not known to cause pandemics^19, 20^. Whereas influenza A viruses can be classified in various subtypes, influenza B viruses have only two antigenically distinct lineages, represented by the prototype viruses B/Victoria/2/1987 (Victoria) and B/Yamagata/16/1988 (Yamagata). Recently, influenza B virus infections have been increasingly recognized as a major cause of influenza-associated morbidity and mortality in high-risk groups, including immunocompromised individuals^7, 17, 18, 21, 22^.

Clinical studies in Japan demonstrated that oseltamivir therapy was less beneficial for patients with influenza B than for those with influenza A virus infections^15, 23, 24^. The virus re-isolation rate in patients receiving antiviral therapy was also significantly higher for influenza B than for influenza A viruses and usually lasting longer^25^. To establish optimal treatment regimens for immunocompromised patients infected with influenza B viruses, animal models are needed to evaluate the efficacy of antiviral treatments. Mouse models are widely used for studying influenza pathogenesis and for evaluating prophylactic and therapeutic strategies; they are convenient to use and robust, and the associated reagents are readily available^26^. Of the available genetically modified, immunodeficient mouse strains with permanently impaired immunity, the severely combined immunodeficient (*scid*) mouse bred from the congenic BALB/c mouse (BALB *scid*) has been most widely used^27, 28^. BALB *scid* mice have a spontaneous mutation in the protein kinase DNA-activated catalytic polypeptide (*Prkdc* ^scid^) gene that severely impairs the generation of B- and T-cell antigen receptors^29, 30^. This leads to very low numbers of functional B- and T-cells and hypogammaglobulinemia, consequently abrogating adaptive immunologic functions^30–32^. The non-obese diabetic (NOD) *scid* mice are derived by backcrossing the BALB *scid* strain onto the NOD/Lt strain background^33, 34^. Compared to BALB *scid* mice, NOD *scid* mice are further impaired in their innate immune mechanisms as a result of defects in their macrophage function, an absence of circulating complement, and decreased natural killer (NK) cell activity, in addition to their lacking of adaptive immunity^33^.

Immunocompromised mice have been used to evaluate treatment options for influenza A virus infections^35, 36^ and to examine the role of T-cell responses during recovery^37^. No similar studies have been conducted for influenza B virus infections. Here, we examine the fundamental biology of influenza B virus infection in immunocompetent BALB/c and immunocompromised BALB *scid* and NOD *scid* mice. To maximize the clinical relevance of the study, we selected three antigenically dominant influenza B viruses representing the Yamagata [B/Massachusetts/2/2012 (MA/12) and B/Phuket/3073/2013 (PH/13)] and Victoria [B/Brisbane/60/2008, (BR/08)] lineages that were included in trivalent and quadrivalent vaccines for the last eight influenza seasons^38^. We investigated whether the difference in immune defects between the mouse strains affected the virus load, duration of replication, or clearance of influenza B viruses. As a proof of concept, we further evaluated the efficacy of intramuscularly administered peramivir, an NAI, against lethal influenza B virus infection in mice.

## Results

### Morbidity and mortality of influenza B viruses in mice

**Figure 1:**
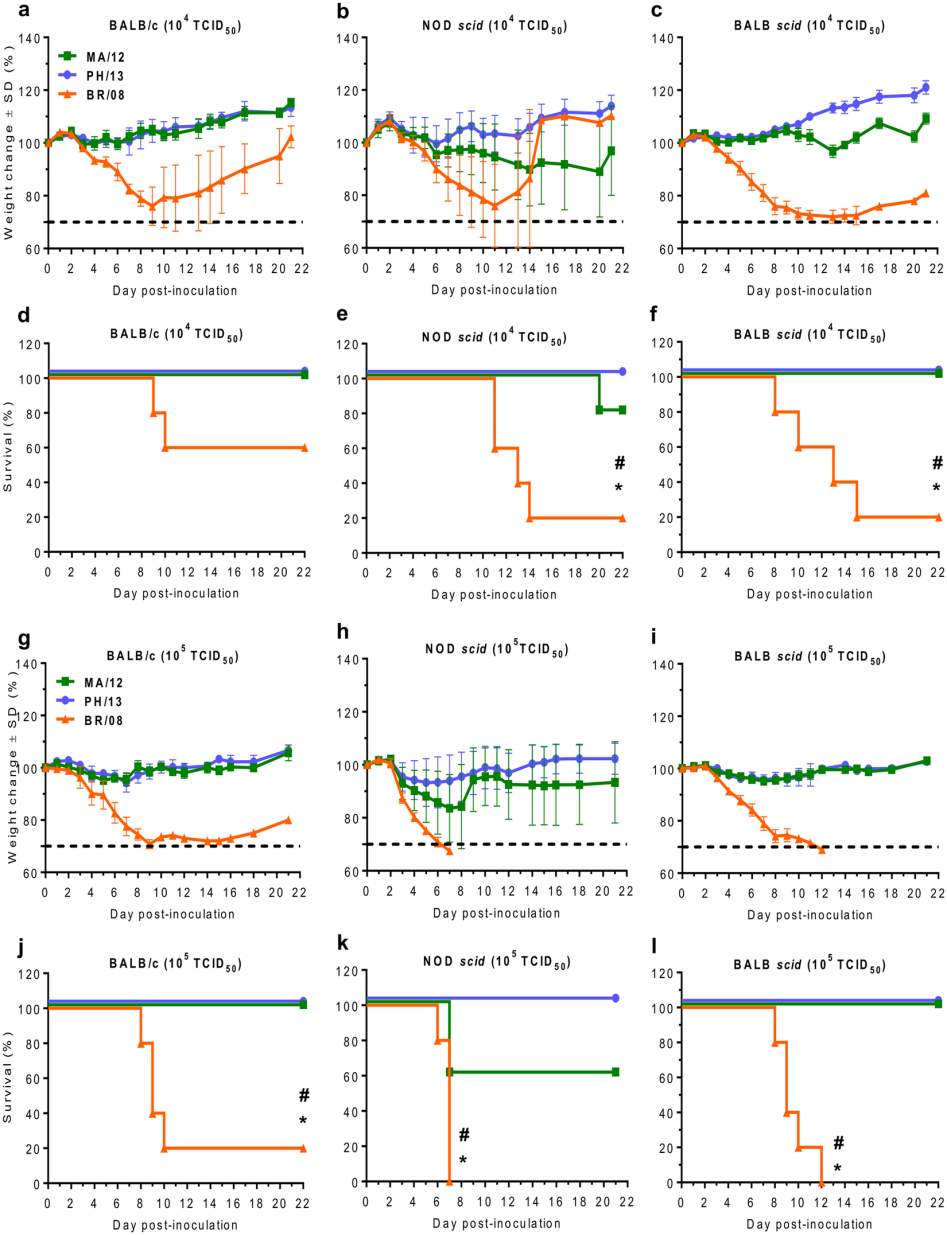
Morbidity and mortality of immunocompetent and immunocompromised mice inoculated with influenza B viruses. Female 6-week-old BALB/c, NOD *scid*, and BALB *scid* mice (*n* = 5/group) were lightly anesthetized with isoflurane and inoculated intranasally with 10^4^ (**a**–**f**) or 10^5^ (**g**–**l**) TCID_50_/mouse of influenza MA/12 (green line), PH/13 (blue line), or BR/08 (orange line) virus. The graphs show the weight loss (**a**–**c**,**g–i**) and survival (**d**–**f**,**j**–**l**) of the inoculated mice, which were monitored and observed daily. Dashed lines indicate endpoint for mortality (30% of initial weight). *P < 0.05, relative to PH/13; ^#^ *P* < 0.05, relative to MA/12, as determined by one-way ANOVA with Bonferroni’s multiple comparison post-test. The probabilities for survival were determined by Kaplan-Meier and log-rank tests, and the differences in weight were analyzed by one-way ANOVA.

The pathogenicity of three influenza B viruses was examined in BALB/c mice and in NOD *scid* and BALB *scid* mice, which have permanently impaired immune status. In immunocompetent BALB/c mice, MA/12 and PH/13 caused no weight change at a dose of 10^4^ TCID_50_, and only marginal weight losses (5%) were detected at 10^5^ TCID_50_ (Fig. 1a,g). No lethality was observed in BALB/c mice inoculated with either Yamagata-lineage virus (Fig. 1d,j). In contrast, Victoria-lineage virus BR/08 induced morbidity in these mice, as manifested by ruffled fur, a hunched back, reduced activity, and weight loss. Maximum weight losses of 24% to 29% were recorded at 9 day post-inoculation (dpi), eventually resulting in the survival of 60% and 20% of immunocompetent mice inoculated with 10^4^ TCID_50_ and 10^5^ TCID_50_ of BR/08, respectively (Fig. 1a,d,g,j).

In immunocompromised mice, 80% of NOD *scid* mice inoculated with 10^4^ TCID_50_ of MA/12 survived infection, as did 60% of NOD *scid* mice inoculated with 10^5^ TCID_50_ (Fig. 1b,e,h,k). PH/13 caused no lethality and induced only minimal weight loss (7% maximum) at 10^5^ TCID_50_ (Fig. 1h,k). In contrast to the Yamagata viruses, BR/08 induced dramatic weight reductions in NOD *scid* mice. Most mice became moribund beyond 2 dpi, resulting in 0% survival with the 10^5^ TCID_50_ virus dose, with a median survival time of 6 days (Fig. 1h,k). With the lower virus dose (10^4^ TCID_50_), survival improved to 20% (Fig. 1b,e). BALB *scid* mice survived infection with MA/12 or PH/13 (100% survival) regardless of the virus dose (Fig. 1c,f,i,l). However, BR/08 infection resulted in survival rates of 20% and 0% at doses of 10^4^ and 10^5^ TCID_50_, respectively. These data showed the variable pathogenicity of the influenza B viruses tested. Specifically, BR/08 consistently induced marked morbidity and was the most lethal virus in both immunocompetent and immunocompromised mice. MA/12 was mildly pathogenic, but only in NOD *scid* mice, whereas PH/13 caused no notable disease in mice of any strain.

### Virus load and duration of influenza B virus replication in mice

**Figure 2:**
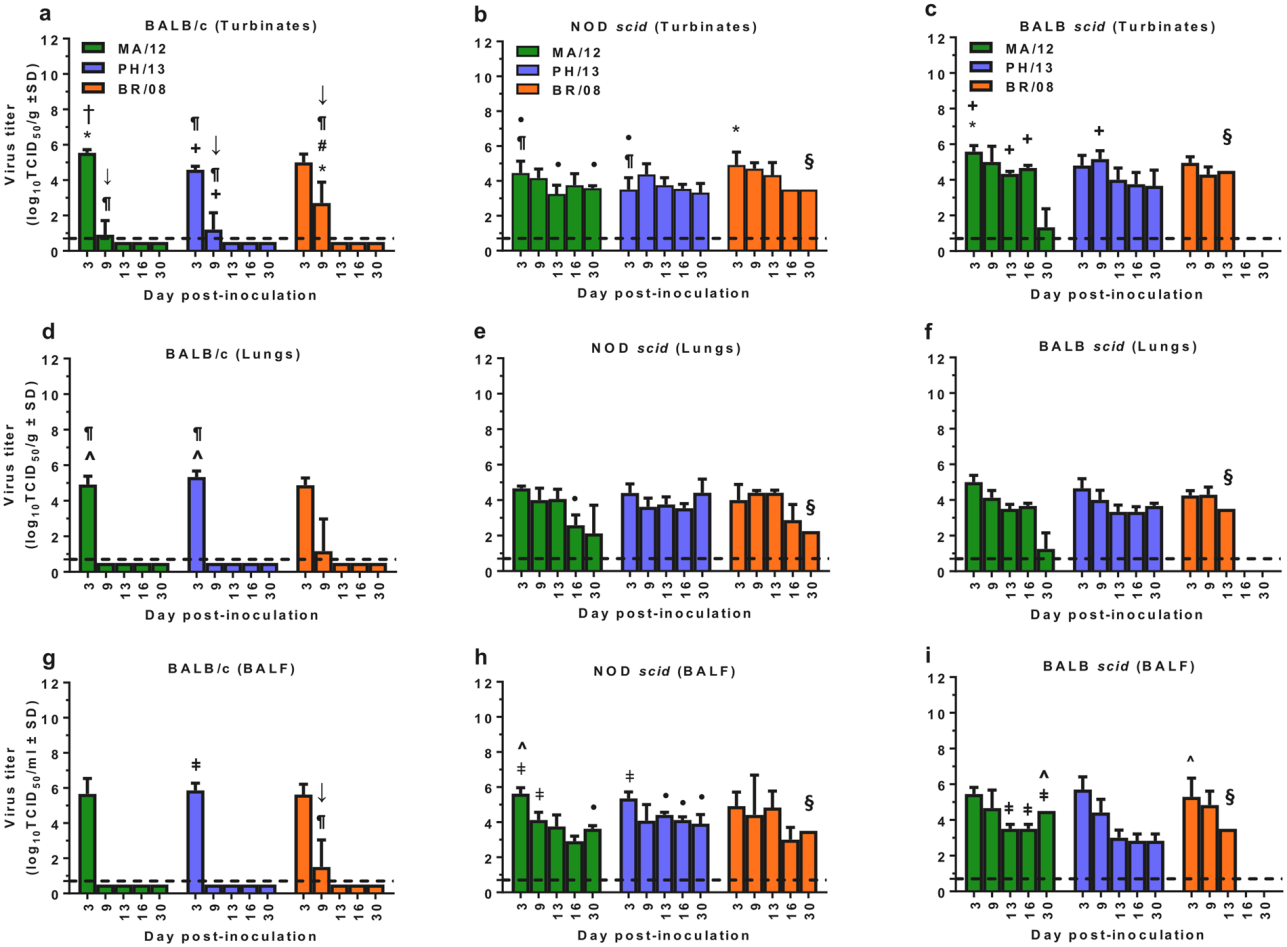
Influenza B virus load and duration of replication in immunocompetent and immunocompromised mice. BALB/c, NOD *scid*, and BALB *scid* mice were lightly anesthetized with isoflurane and inoculated intranasally with 10^4^ TCID_50_/mouse of influenza PH/13 (blue bars), MA/12 (green bars), or BR/08 (orange bars) virus. Virus titers were determined in the nasal turbinates (**a**–**c**), lungs (**d**–**f**), and BALF (**g**–**i**) of mice (*n* = 3/group/time-point) at 3, 9, 13, 16, and 30 dpi by TCID_50_ assays in MDCK cells. The bars represent the mean virus titers ± SD (log_10_TCID_50_/mL) for each time-point. Dashed lines indicate the minimum level of virus detection (0.75 log_10_TCID_50_/mL). *P < 0.05, relative to PH/13; ^#^ *P* < 0.05, relative to MA/12; ^†^ *P* < 0.05, relative to BR/08; ^+^ *P* < 0.05 for virus titers in nasal turbinates versus lungs; ^ǂ^ *P* < 0.05 for virus titers in nasal turbinates versus BALF; ^*P* < 0.05 for virus titers in lungs versus BALF; ^¶^ *P* < 0.05 for virus titers in BALB/c versus NOD *scid* mice; ^↓^ *P* < 0.05 for virus titers in BALB/c versus BALB *scid* mice; and ^•^ *P* < 0.05 for virus titers in NOD *scid* mice versus BALB *scid* mice, as determined by one-way ANOVA with Bonferroni’s multiple comparison post-test. ^§^Detection in one mouse only.

Virus load and replication efficiency contribute to influenza virus pathogenicity in humans and mammalian animal models^39–41^. Accordingly, we explored the kinetics and duration of influenza B virus replication in the three mouse strains. The replication of all the viruses studied was restricted to respiratory tissues, with none detected in extra-pulmonary organs (data not shown). In BALB/c mice, all influenza B viruses attained high titers in the upper and lower respiratory tract (URT and LRT) at 3 dpi (Fig. 2a,d,g). At this time-point, significantly higher titers (*P* < 0.05) were found in the nasal turbinates of mice infected with MA/12 than in those of mice infected with PH/13 or BR/08 (5.5 versus 4.6 or 5.0 log_10_TCID_50_/mL) (Fig. 2a), although the respective titers of these viruses in the lungs and bronchoalveolar lavage fluid (BALF) of mice were almost equivalent (Fig. 2d,g). PH/13 replicated to higher titers in the LRT than in the URT (5.6 versus 4.6 log_10_TCID_50_/mL, *P* < 0.05). By 9 dpi, the viral titers in the nasal cavities of mice infected with BR/08 were significantly higher than those in mice infected with the Yamagata viruses (2.6 versus 0.7 and 0.9 log_10_TCID_50_/mL, *P* < 0.05) (Fig. 2a), and only BR/08 was additionally recovered from the LRT (Fig. 2a,d,g). Thus, BR/08 replicated longer than the Yamagata viruses in BALB/c mice.

In NOD *scid* mice, all influenza B viruses replicated until 30 dpi. Despite the variable mortality observed (Fig. 1), viral titers were comparable for the three viruses at most time-points (range, 3.3–4.9 log_10_TCID_50_/mL in nasal turbinates, 2.6–4.6 log_10_TCID_50_/mL in lungs, and 2.9–5.6 log_10_TCID_50_/mL in BALF) (Fig. 2b,e,h). BR/08 titers in the nasal turbinates at 3 dpi were significantly higher than those for PH/13 (4.9 versus 3.5 log_10_TCID_50_/mL, *P* < 0.05) (Fig. 2b). In BALB *scid* mice, prolonged replication in the URT and LRT was observed for all viruses (up to 30 dpi) and was similar to that observed in NOD *scid* mice. At 13 dpi, mice inoculated with BR/08 started to succumb to infection, and no animals were available for sample collections at 16 or 30 dpi. The viral titers (range, 3.8–5.0 log_10_TCID_50_/mL in nasal turbinates, 3.3–5.0 log_10_TCID_50_/mL in lungs, 2.8–6.0 log_10_TCID_50_/mL in BALF) were also comparable at most time-points (Fig. 2c,f,i).

Given the differences in the immune statuses, we compared the virus replication kinetics between the NOD *scid* and BALB *scid* mice. Titers in the URT of BALB *scid* mice inoculated with the Yamagata viruses were slightly higher than those in NOD *scid* mice, whereas almost equivalent virus titers were obtained in the LRT, regardless of the mouse strain (Fig. 2b,e,h vs. Fig. 2c,f,i). This trend was not observed in BR/08-inoculated immunocompromised mice. Thus, we saw no major differences in the growth kinetics and duration of viral shedding of influenza B viruses in the two immunocompromised mouse models.

### Inflammatory leukocyte infiltration in the BALF of mice inoculated with influenza B viruses

**Figure 3:**
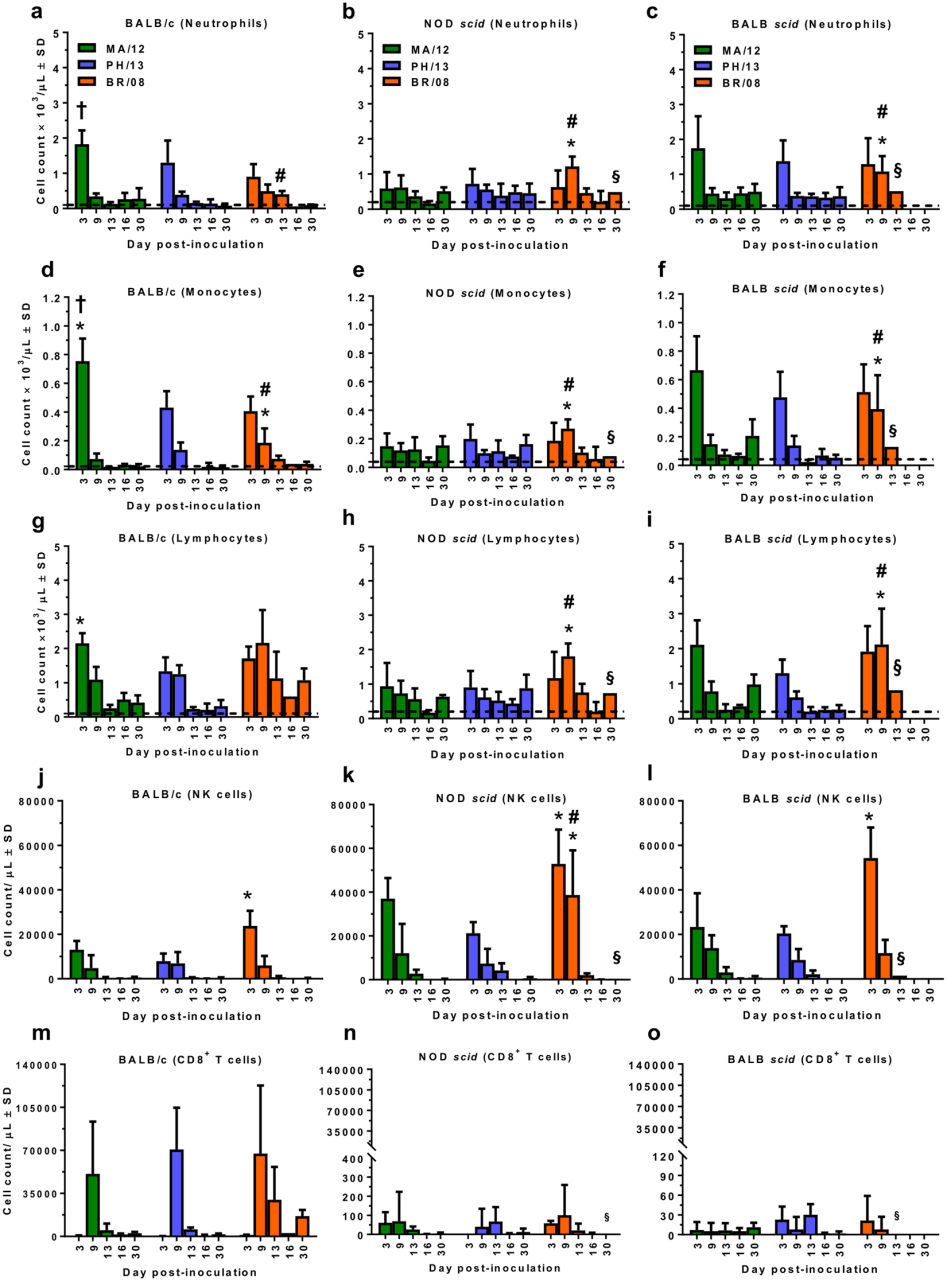
Induction of virus-induced inflammatory correlates in BALF of immunocompetent and immunocompromised mice inoculated with influenza B viruses. BALB/c, NOD *scid*, and BALB *scid* mice were inoculated with viruses as described in the legend for Fig. 2. Inflammatory cell counts [neutrophils (**a**–**c**), monocytes (**d**–**f**), and lymphocytes (**g**–**i**)] were determined in the BALF of virus-inoculated mice at 3, 9, 13, 16, and 30 dpi by automatic cell counter and lymphocyte subpopulations [NK (**j**–**l**) and CD8^+^ T (**m**–**o**) cells] were differentiated by flow cytometry. Bars represent mean values ± SD (*n* = 3/group/time-point). ^*^ *P* < 0.05, relative to PH/13; ^#^ *P* < 0.05, relative to MA/12; and ^†^ *P* < 0.05, relative to BR/08, as determined by one-way ANOVA with Bonferroni’s multiple comparison post-test. ^§^Detection in one mouse only. Dashed line indicates the cell counts in control mice inoculated with sterile 1× PBS and sacrificed at 6 dpi.

Host immune responses can influence the outcome of influenza virus infection in humans and mammalian animal models^39, 42–45^. We assessed whether the immune responses induced by influenza B virus differed in immunocompetent and immunocompromised mice and whether these corresponded to the morbidity and mortality observed. In BALB/c mice, the BALF showed early infiltration of neutrophils, monocytes, and lymphocytes at 3 dpi, most significantly (*P* < 0.05) in those mice inoculated with MA/12 (Fig. 3a,d,g). By 9 dpi, virus-induced cell infiltrates had largely subsided to levels close to the detection limit (dashed lines), except in mice inoculated with BR/08 (Fig. 3a,d,g). At 13 dpi, significantly higher neutrophil and monocyte cellularity (*P* < 0.05) was maintained in BR/08-inoculated mice, as compared to mice inoculated with Yamagata viruses (Fig. 3a,d). With respect to the subpopulations of lymphocytic immune cells, BR/08 also elicited significantly higher levels of NK cells (*P* < 0.05) at 3 dpi than did PH/13 (Fig. 3j). B-cell, CD8^+^, and CD4^+^ T-cell immune responses to the three viruses were comparable in BALB/c mice (Fig. 3m and Supp. Fig. 1a,d).

In NOD *scid* mice, influenza B virus infections induced modest recruitment of inflammatory immune cells, reflecting the defective innate immune responses of these animals. At 9 dpi, neutrophils, monocytes, and lymphocytes (mainly NK cells) were persistently elevated in mice inoculated with BR/08, as compared to animals inoculated with MA/12 or PH/13 (*P* < 0.05) (Fig. 3b,e,h,k). Moreover, higher infiltration of BR/08-induced NK cells in NOD *scid* than in BALB/c mice was observed at this time-point (Fig. 3k versus 3j), suggesting ample number of NK cells in response to influenza B virus infection despite reportedly having reduced activity^33^. Virus-induced cellular infiltration was more pronounced in BALB *scid* than in NOD *scid* mice, and the patterns were similar to those observed in BALB/c mice (Fig. 3c,f,i). At 3 dpi, NK-cell recruitment induced by BR/08 was also significantly higher than that induced by PH/13 (*P* < 0.05) (Fig. 3l). Although the cellular recruitment induced by MA/12 and PH/13 was largely reduced by 9 dpi, the neutrophil, monocyte, and lymphocyte responses induced by BR/08 were still significantly greater at 9 dpi than those induced by the Yamagata viruses (*P* < 0.05 to *P* < 0.01). The levels of NK cells elicited at 9 dpi were comparable to those in BALB/c mice but were significantly lower than those in NOD *scid* mice (Fig. 3l versus 3j,k). Both T- and B-cell recruitment due to influenza B virus infections in the NOD *scid* and BALB *scid* mice were marginal relative to that detected in BALB/c mice, confirming the impaired adaptive immune responses in these animals (Fig. 3n,o, and Supp. Fig. 1b,c,e,f). Thus, immune-response induction in the immunocompromised mice, particularly NOD *scid* mice, was relatively low compared to that in BALB/c mice, except in BR/08-inoculated animals. Of the two immunocompromised mouse strains, BALB *scid* mice exhibited the most cellular infiltration in their BALF in response to influenza B virus infection. Importantly, BR/08 consistently induced more robust and sustained inflammatory cell infiltrates in both immunocompromised mouse strains than did the Yamagata viruses, suggesting that immunopathology contributes to the higher pathogenicity of BR/08.

A panel of HI assays was used to confirm that the defective T- and B-cell responses in NOD *scid* and BALB *scid* mice did not also promote neutralizing antibody production. BALB/c mice developed anti-HA antibodies against the respective homologous viruses (Table 1), whereas none of the immunocompromised animals did, emphasizing the lack of adaptive immune responses resulting from their inability to produce functionally mature T- and B-cells^29, 31, 32^.

**Table 1:** Anti-HA antibody responses in immunocompetent and immunocompromised mice and susceptibility of influenza B viruses to NAIs in NA enzyme inhibition assay.

Influenza B virus	Genetic lineage	Mouse strain^a^			Susceptibility to NAIs (Mean IC_50_ ± SD, nM)^b^		
		BALB/c	NOD *scid*	BALB *scid*	Oseltamivir	Zanamivir	Peramivir
MA/12	Yamagata	320	<^c^	<	8.2 ± 2.9	1.3 ± 0.2	0.5 ± 0.1
PH/13	Yamagata	1080	<	<	9.6 ± 2.0	1.8 ± 0.4	0.5 ± 0.1
BR/08	Victoria	640	<	<	16.0 ± 2.9	2.9 ± 0.6	1.1 ± 0.2

^a^BALB/c, NOD *scid* and BALB *scid* mice were intranasally inoculated with 10^4^ TCID_50_/mouse of influenza B virus. Serum samples were obtained from the surviving mice at 30 dpi and mean HI titers were determined with 0.5% tRBC and expressed as the reciprocal of the highest dilution of serum that inhibited 8 HA units of virus (e.g., as 80 versus 1:80).

^b^Concentration of NAI that reduced viral NA activity by 50% relative to NA activity without inhibitor. Values represent the mean IC_50 _± SD from two independent experiments performed in triplicate.

^c^HI titers were below the assay limit of detection (HI titer of 40).

### Comparative histopathology in mice inoculated with influenza B viruses

**Figure 4:**
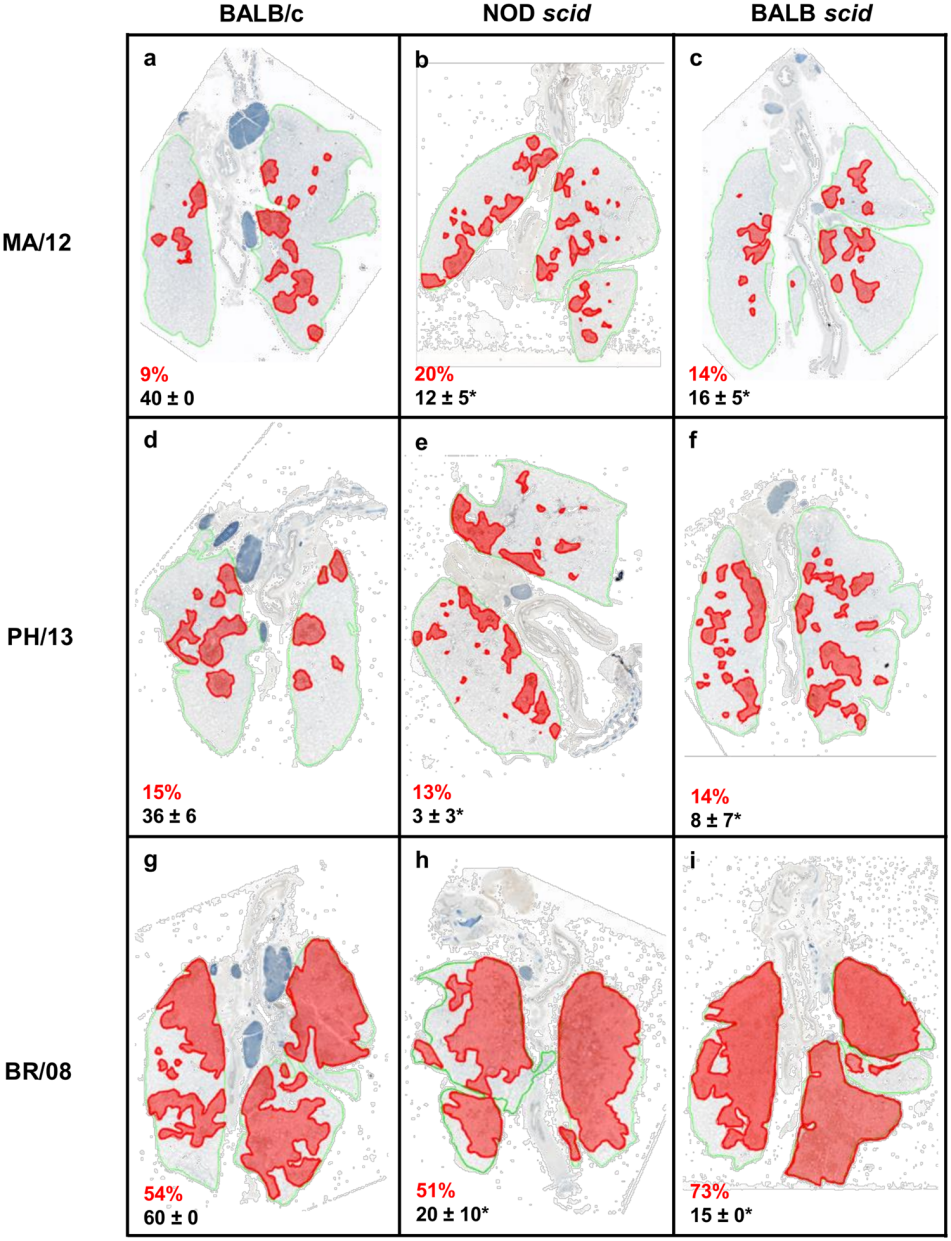
Histopathologic changes in the lungs of immunocompetent and immunocompromised mice inoculated with influenza B viruses. BALB/c, NOD *scid*, and BALB *scid* mice were inoculated with viruses as described in the legend for Fig. 2. Pulmonary lesions were evaluated at 6 dpi (*n* = 4/virus/mouse strain). The panels show the extent of virus infection and histopathology of influenza MA/12, PH/13, and BR/08 viruses in the lungs of immunocompetent BALB/c (**a**,**d**,**g**) and immunocompromised NOD *scid* (**b**,**e**,**h**) and BALB *scid* (**c**,**f**,**i**) mice. A representative section is shown for each virus and mouse strain. The green lines designate total lung areas measured; red-shaded areas designate bronchioles/alveoli with active virus infection (i.e., containing antigen-positive epithelial cells). The average percentage of the total lung field containing antigen-positive epithelial cells are shown in red texts and the average severity score ± SE for inflammation are shown in black texts. *P < 0.05, relative to BR/08 scores, as determined by one-way ANOVA with Bonferroni’s multiple comparison post-test.

Inoculation of the Yamagata viruses in the immunocompetent and immunocompromised mouse strains showed a similar extent of virus spread (Fig. 4a,b,c,d,e,f); BR/08 virus infection in all mice did not also differ markedly (Fig. 4g,h,i). While both MA/12 and PH/13 induced small, localized lesions, BR/08 affected more than half of the lung area. However, based on alveolar lesion scores (Fig. 4, black texts), the overall severity of inflammatory cell infiltrates and lesions was significantly greater in BALB/c than in NOD *scid* and BALB *scid* mice, including in BR/08-inoculated groups (Fig. 4a,d,g versus 4b,e,h and c,f,i) (*P* < 0.05).

Whereas virus antigen was generally restricted to the peribronchiolar alveoli in immunocompromised mice inoculated with Yamagata viruses, more extensive areas were affected by BR/08. The extent of infection and the overall severity of pulmonary lesions were markedly increased in BALB/c, NOD *scid*, and BALB *scid* mice inoculated with BR/08 (Fig. 4g–i), as compared to mice inoculated with MA/12 or PH/13 (Fig. 4a–f). The more aggressive and extensive spread of BR/08 in the lungs of mice of all three strains is reflected in the increased percentage of lung fields that contained cells positive for virus antigen and in the marked reduction in type II pneumocytes positive for surfactant protein C. Furthermore, alveolar protein exudates and hyaline membrane formation, which signify severe damage to the alveolar capillary membranes, were most severe in the BALB *scid* mice infected with BR/08.

### Inflammatory cytokines and chemokines in the BALF of mice inoculated with influenza B viruses

**Figure 5:**
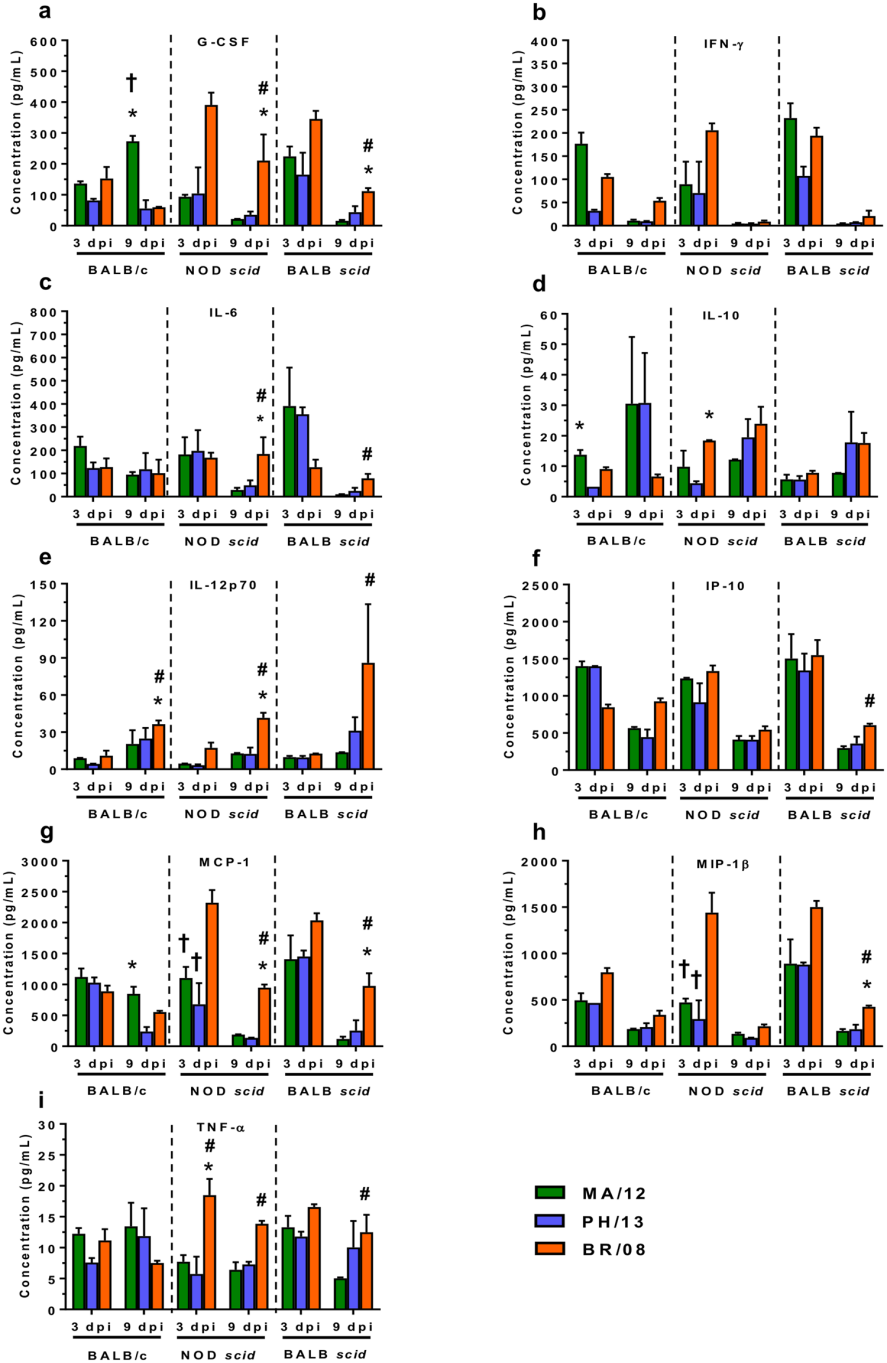
Induction of pulmonary cytokine/chemokine responses in immunocompetent and immunocompromised mice inoculated with influenza B viruses. BALB/c, NOD *scid*, and BALB *scid* mice were inoculated with viruses as described in the legend for Fig. 2. The expression levels of 25 cytokines and chemokines were assayed in lung homogenates at 3 and 9 dpi. Bars represent mean values ± SE (*n* = 5–6/group/time-point). ^*^ *P* < 0.05, relative to PH/13; ^#^ *P* < 0.05, relative to MA/12; and ^†^ *P* < 0.05, relative to BR/08, as determined by one-way ANOVA with Bonferroni’s multiple comparison post-test.

The cytokine response can affect the severity of influenza disease in both humans and mammalian animal models^39, 46^. We analyzed the levels of pulmonary pro- and anti-inflammatory cytokines and chemokines at 3 and 9 dpi (Fig. 5 and Supp. Fig. 2). These specific time-points reflect the initiation of innate and adaptive immune responses^47^. The concentrations of granulocyte colony-stimulating factor (G-CSF), interferon-γ (IFN-γ), interleukin 6 (IL-6), IL-10, IL-12p70, IFN-γ–inducible protein 10 (IP-10), monocyte chemotactic protein 1 (MCP-1), macrophage inflammatory protein 1β (MIP-1β), and tumor necrosis factor α (TNF-α) were substantially increased (*P* < 0.05) in virus-inoculated BALB/c mice, compared to those in uninfected controls (Supp. Fig. 2). At 3 dpi, the levels of IFN-γ, IL-6, IL-10, MCP-1, and TNF-α in BALB/c mice infected with MA/12 (Fig. 5b–d,g,i) were considerably higher than in animals inoculated with PH/13 or BR/08; however, only the IL-10 elevation was significant (*P* < 0.05) relative to that observed with PH/13 infection (Fig. 5d). By 9 dpi, MA/12 elicited a G-CSF level (*P* < 0.05) approximately 5-fold higher than that observed with PH/13 and BR/08 (Fig. 5a). With respect to BR/08, G-CSF, IL-12p70, and MIP-1β were substantially elevated at 3 dpi, although their levels were not significantly higher than those induced by the Yamagata viruses (Fig. 5a,e,h). By 9 dpi, the levels of IP-10, MIP-1β, IFN-γ, and IL-12p70 were also higher, but only the IL-12p70 elevation was significant (*P* < 0.05) (Fig. 5a,d,e,g), whereas the level of IL-10 remained low at 9 dpi (Fig. 5f).

In NOD *scid* mice infected with BR/08, the concentrations of IL-10, IL-12p70, MCP-1, MIP-1β, and TNF-α in the lungs at 3 dpi were significantly elevated when compared with those in mice infected with PH/13 or MA/12 (*P* < 0.05) (Fig. 5d,e,g,h,i). At 9 dpi, IL-12p70, MCP-1, and TNF-α remained highly elevated with the addition of G-CSF and IL-6 (Fig. 5a,c). In BALB *scid* mice, the levels of G-CSF, IL-6, IL-12p70, IP-10, MCP-1, MIP-1β, and TNF-α were also significantly higher at 9 dpi in BR/08-inoculated animals than in those inoculated with Yamagata viruses (*P* < 0.05). Thus, BR/08 initiated markedly higher and sustained induction of pro-inflammatory cytokines, which could promote deleterious immunopathology. Moreover, the cytokine and chemokine elevations in both immunocompromised mouse strains were similar but ultimately differed from that in the immunocompetent mice.

### Efficacy of peramivir against lethal challenge with BR/08 in mice

We next explored the therapeutic effect of the NAI peramivir on the survival of BALB/c and BALB *scid* mice when treatment was initiated 24 h after lethal challenge with BR/08. Our treatment regimen, in which peramivir was administered every other day, was based on the available mouse pharmacokinetics data, which suggest that the half-life of peramivir is more than 24 h^48^. Phenotypic assays revealed that all the influenza B viruses tested were susceptible to currently available NAIs (Table 1). In particular, BR/08 demonstrated susceptibility to oseltamivir, zanamivir, and peramivir with IC_50_ values of 16.0, 2.9, and 1.1 nM, respectively.

**Figure 6:**
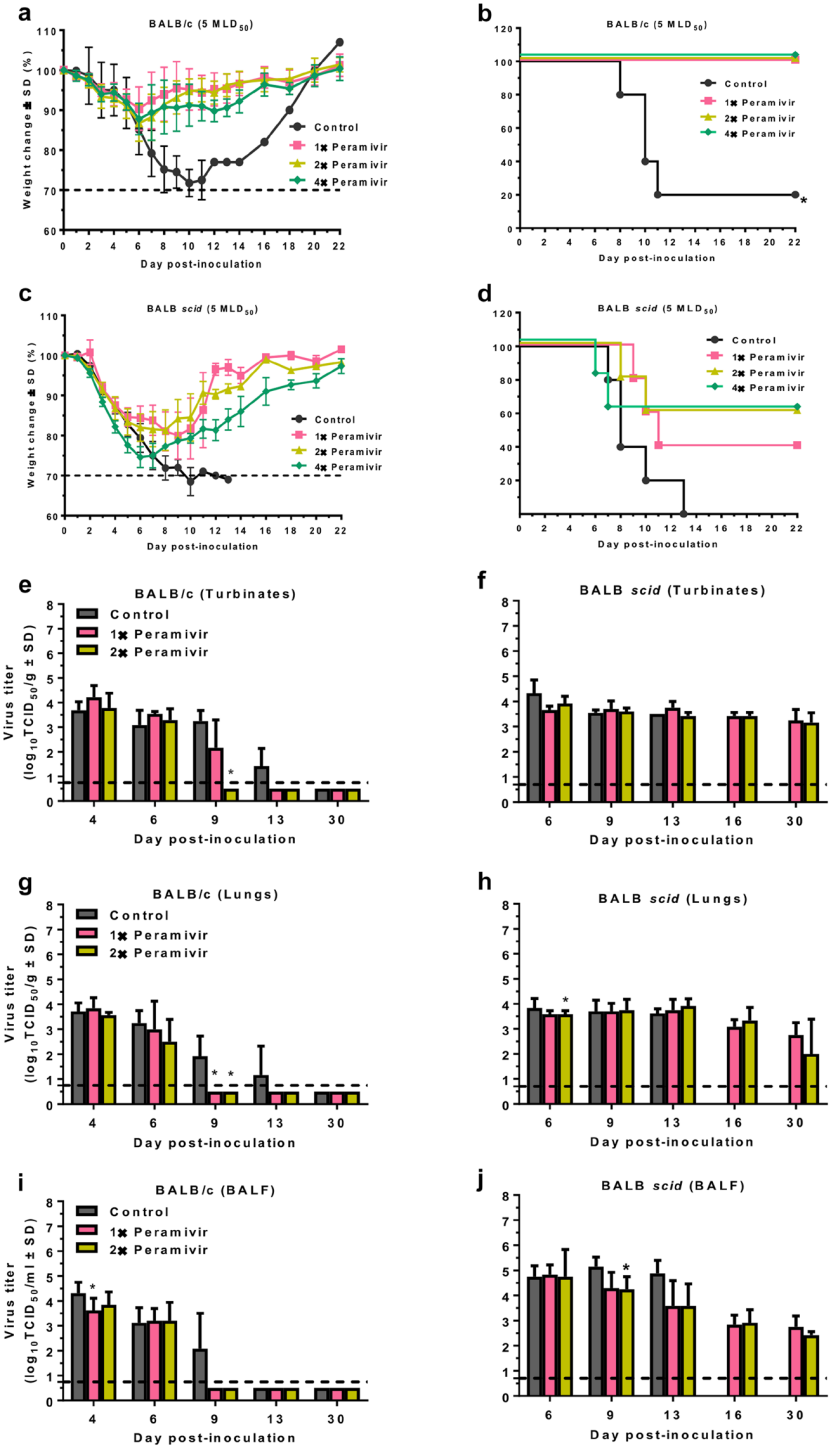
Efficacy of peramivir treatment on survival of immunocompetent and immunocompromised mice inoculated with influenza BR/08 virus. BALB/c and BALB *scid* mice (*n* = 5/group) were lightly anesthetized with isoflurane and inoculated with 5 MLD_50_ (2.5 × 10^5^ TCID_50_/mouse) of influenza BR/08 virus. The NAI peramivir was administered by IM injection at a dose of 75 mg/kg/day (0.1 mL/mouse) once a day in a single-dose (1×: +24 hpi), double-dose (2×: +24 and +72 hpi), or four-dose (4×: +24, +72, +120, and +168 hpi) regimen. Control animals received IM injection of sterile RNAse-free water in a four-dose regimen (4×: +24, +72, +120, and +168 hpi). The graphs show the weight loss (**a**,**c**) and survival (**b**,**d**) of inoculated mice, which were monitored and observed daily, up to 30 dpi. The bar charts show the effect of peramivir treatment (1×: +24 hpi or 2×: +24 and +72 hpi) on virus titers in nasal turbinates (**e**,**f**), lungs (**g**,**h**), and BALFs (**i**,**j**) of BALB/c and BALB *scid* mice (*n* = 3/group/time-point) inoculated with BR/08 virus at 4, 6, 9, 13, 16 and 30 dpi. Virus titers were determined by TCID_50_ assays in MDCK cells. *P < 0.05, compared between control and peramivir-treated mice inoculated with BR/08 virus. Probabilities for survival were determined by the Kaplan-Meier and log-rank tests, and the differences in weight were analyzed by one-way ANOVA.

In BALB/c, control animals progressively lost weight, and 80% of the mice succumbed to virus infection between 8 and 11 dpi (Fig. 6a,b). In contrast, peramivir conferred complete protection against lethality, whether it was administered in one (1×), two (2×), or four (4×) doses, with maximum mean weight loss of 10% to 14%. In BALB *scid*, control animals rapidly lost weight and did not survive the infection beyond 13 dpi (100% mortality rate, mean survival of 8 days) (Fig. 6c,d). Single injection of peramivir prevented death in 40% of BALB *scid* mice whereas 2×- and 4×-dose afforded 60% protection from lethal infection.

To determine whether peramivir treatment affected virus replication, we determined the virus titers at 4, 6, 9, 13, 16, and 30 dpi in the URT and LRT of mice treated with a 1× or 2× dose regimen. In BALB/c mice, the viral titers did not significantly differ between the 1× and 2× dose groups (Fig. 6e,g,i). Peramivir treatment resulted in a trend of decreasing virus replication in the nasal cavities and lungs relative to that seen in control animals, and at 9 dpi this trend was more pronounced in animals treated with the 2× dose regimen (*P* < 0.01) (Fig. 6e). At 13 dpi, both treatment groups had already cleared the virus, whereas infectious viruses could still be recovered from the URT and LRT of control animals (Fig. 6e,g). In BALB *scid* mice, peramivir treatment reduced the virus titers in the BALF, when compared to that in control animals, particularly at 9 dpi in the 2× dose group (*P* < 0.05), but did not reduce the titers in the nasal turbinates or lungs (Fig. 6f,h,j). Thus, peramivir treatment has the potential to reduce virus replication in BALB *scid* mice, but neither the 1× nor 2× dose regimen was sufficient to facilitate complete virus clearance.

To monitor the emergence of resistant variants after peramivir treatment, we isolated RNA from viruses recovered from individual plaques produced by lung samples collected at 9, 16 and 30 dpi. Sequence analysis of the NA glycoprotein identified a plaque clone carrying G145E substitution (B numbering) from a BALB *scid* mouse lung in the 2× dose regimen collected at 9 dpi. Compared to the parental virus, this clone had an increased 50% inhibitory concentration (IC_50_), reducing susceptibility to peramivir by 4.3-fold. Although still considered as normal inhibition based on WHO guidelines^49^, it is well within the borderline for reduced susceptibility (>5-fold), highlighting its consequence on antiviral resistance as previously reported^50, 51^. No other differences between the NA sequence of the challenge BR/08 and that of the viruses isolated from the lungs of peramivir-treated BALB/c and BALB *scid* mice associated with NAI-resistance were detected (data not shown).

## Discussion

There have been few preclinical studies of the biological properties of influenza B viruses as a result of their limited host range and their restricted replication in animal models. Various immunocompromised mouse strains have been used to model human infectious diseases and evaluate adaptive and innate immune responses^27, 28^. Here, we used two mouse strains with permanently impaired immunity (NOD *scid* and BALB *scid* mice) to study influenza B virus infection and the efficacy of peramivir against lethal virus challenge. The three influenza B viruses exhibited different degrees of pathogenicity in immunocompromised mice, with the BR/08 virus causing significantly more morbidity and lethality. Such pathogenesis was associated more with sustained induction of innate immune cell infiltrates and immune-mediated lung pathology than with enhanced virus replication efficiency. Importantly, both mouse strains supported prolonged virus replication, such as typically observed in immunocompromised hosts^2, 4, 5^. Despite this, we detected no virus antigen–specific antibodies, which is consistent with the absence of functional B- and T-cell responses in these mice^29–33^. Moreover, repeated doses of peramivir rescued BALB *scid* mice from severe morbidity and mortality caused by lethal challenge with BR/08.

NOD *scid a*nd BALB *scid* mice infected with influenza B viruses generally developed mild to moderate influenza-like disease, except in BR/08-infected animals. Influenza B viruses demonstrated more prolonged replication in NOD *scid* and BALB *scid* mice than in BALB/c mice, which is consistent with observations in transiently immunosuppressed and permanently immunocompromised mice infected with influenza A viruses^35, 36^. However, the extended virus replication was not accompanied by severe morbidity, and immunocompromised mice were largely asymptomatic for much of the infection period. Prolonged asymptomatic virus propagation in immunocompromised patients increases the risk of virus transmission to close contacts and the development of antiviral drug-resistance among patients undergoing treatment^5^. NOD *scid* mice inoculated with MA/12 or BR/08 manifested moderately greater morbidity than BALB *scid* mice inoculated with either virus. However, the viral titers appeared to be similar at most time-points. Therefore, the genetic variations between the NOD *scid* and BALB *scid* mice did not result in marked differences in their susceptibility to influenza B virus infection or in the severity of the resulting disease.

Inflammatory cell responses against influenza B virus infection were initiated in the immunocompromised mice, albeit with considerably lower intensity in NOD *scid* mice than those in immunocompetent mice. Interestingly, NOD *scid* mice demonstrated enhanced NK cell activity upon infection which is consistent with the elevations noted against herpes simplex virus type 1^52^ or poly I:C stimulation^33^. Together, these results suggest that NK cells contribute largely to the initiation of chemokine/cytokine production in NOD *scid* mice during infection. Regardless, elicited immune responses in both immunocompromised mice did not regulate virus persistence. The lung pathology in these immunocompromised mice was also generally milder than that in BALB/c mice. Together, these factors helped define the generally mild clinical disease in these animals. Unlike NOD *scid* mice, BALB *scid* mice have intact, normally functioning innate immune cells (i.e., neutrophils, monocytes, NK cells, and macrophages)^31, 32^. The finding that the MA/12 virus exhibited morbidity and mortality in NOD *scid*, but not in BALB *scid* and BALB/c mice, implies a potentially important role of the innate immune system in protecting mice against low pathogenic influenza B virus. However, the extended virus persistence in BALB *scid* mice confirms that an intact innate immune system alone is insufficient for efficient viral clearance. Whereas innate immune responses initiate inflammation, restrict virus replication, and activate adaptive immune responses^53^, it is the virus-specific adaptive immune responses (e.g., antibodies and CD4^+^ and CD8^+^ T-cells) that ultimately control virus propagation and eliminate virus-infected cells^54^. Thus, the lack of neutralizing antibodies and T-cell responses in NOD *scid* and BALB *scid* mice accounted for the virus persistence.

Our data revealed that BR/08 consistently induced severe morbidity and lethality in the three mouse strains used in this study, providing evidence that influenza B viruses can cause lethal infection without prior adaptation^17, 55^. Infection with BR/08 was also associated with severe disease in pregnant ICR mice^56^ and in ferrets, in which it established an efficient LRT infection with a high viral burden^57, 58^. Although BR/08 tend to replicate longer than MA/12 and PH/13 in BALB/c mice, it did not produce significantly different viral titers than those obtained with the Yamagata viruses (including in NOD *scid* and BALB *scid* mice), indicating that other factors rather than virus load could be responsible for its pathogenicity.

BR/08-induced morbidity and mortality correlated with the virus spread and extent of alveolar damage in all mouse strains. The immunocompromised mice generally succumbed to BR/08 virus infection by 13 dpi, whereas MA/12 and PH/13 virus-inoculated animals recovered. The extent of infection and the loss of type II pneumocytes was significantly increased in NOD *scid* and BALB *scid* mice infected with BR/08, despite the viral titers being comparable to those of the Yamagata viruses. Moreover, the increased alveolar proteinosis and hyaline membrane formation associated with BR/08 infection are indicative of more extensive and severe damage to the alveolar capillary membranes and are consistent with the severe damage to bronchioles and alveoli that this virus causes in ferrets^58^.

Influenza virus–associated mortality might also result from an exaggerated immune response or impaired virus clearance^59, 60^. Given the extended replication and immunopathology of BR/08, the sustained immune cell infiltration was predictable. The immune responses to BR/08 in NOD *scid* and BALB *scid* mice predominantly involved NK cells, suggesting that this sustained NK-cell activity mediated immunopathology, as opposed to providing protective role. NK cells did not protect against lethal doses of influenza A virus in *scid* mice^37^. Accordingly, the sustained release of endogenous pro-inflammatory G-CSF, IL-6, IL-12, MCP-1, and TNF-α in NOD *scid* and BALB *scid* mice inoculated with BR/08 at later time-points may also be a determinant of altered pathogenicity. Each of these chemotactic proteins plays a role in recruiting immune leukocytes, including NK cells, but their aberrant and excessive overexpression also decreases the survival of humans or animals infected with highly pathogenic A(H5N1) viruses^39, 43–46^. With the pulmonary damage induced by BR/08, the prolonged inflammation mediated by continued NK cell activity, together with the overexpression of chemotactic proteins could delay recovery from infection and the initiation of alveolar repair mechanisms, which would be detrimental to survival. Therefore, extensive virus spread with severe pulmonary pathology, sustained NK cell responses, and extended induction of pro-inflammatory cytokines and chemokines determined the BR/08 virus-associated pathogenicity rather than severe virus burden.

Currently, peramivir (Rapivab®) is the only FDA-approved parenteral NAI indicated for treating acute uncomplicated influenza in patients aged 18 years or older^61^. Preclinical studies in mice showed that IM peramivir injections are effective against seasonal influenza A^48, 62–65^ and against highly pathogenic A(H5N1) viruses^66^. A single intravenous peramivir injection reduced viral titers and disease signs in ferrets and cynomolgus macaques infected with influenza B viruses^67^. However, antiviral treatment has never been evaluated in immunocompromised animals infected with influenza B virus. Our data show that BALB *scid* mice responded to peramivir therapy but that at least two doses (as compared to the single dose recommended in humans)^61^ were needed to reduce morbidity and promote 60% survival against lethal challenge with BR/08. Although we observed a disease-inhibitory effect with peramivir treatment based on the survival and morbidity data, we did not see efficient suppression of virus replication in BALB *scid* mice. We hypothesize that peramivir treatment initially restrained virus replication, providing a survival window that enabled the mice to withstand lethal infection. However, it is plausible that a proportion of residual virus rebounded from restrained replication after antiviral administration was halted, as observed in immunocompromised mice infected with influenza A virus after oseltamivir treatment^36^. Isolation of a virus clone bearing peramivir resistance-associated NA substitution at position 145^50, 51^ demonstrated that BALB *scid* mice could be a suitable model to study the emergence of drug-resistant variants under antiviral pressure in immunocompromised host. We cannot rule out the possibility that more NAI-resistant variants would be observed with prolonged drug administration or that minor populations of resistant variants could be detected by using next-generation sequencing.

Overall, our data show that immunocompromised mice exhibited prolonged influenza B virus replication and altered host immune induction and that the infection responded to antiviral therapy. Additional studies with these models to evaluate the efficacy of currently available or investigational anti-influenza compounds and vaccines are needed, including evaluations of administration methods, dose frequencies, and combination therapies. Further investigations of the efficacy of combinations of virus-targeted and host-targeted antiviral treatments (immunomodulatory drugs) during severe influenza A and B virus infections in immunocompromised patients are warranted. In summary, our findings increase our basic understanding of how to better manage influenza virus infections in these high-risk groups.

## Materials and Methods

### Ethics statement

All protocols and procedures followed in the study were approved by the St. Jude Animal Care and Use Committee (IACUC) and complied with the policies of the National Institutes of Health and the Animal Welfare Act. All animal experiments were conducted at St. Jude Children’s Research Hospital (Memphis, TN) in accordance with applicable laws and guidelines and after approval by the Institutional Animal Care and Use Committee.

### Viruses and cells

The influenza B viruses [MA/12 (Yamagata lineage), PH/13 (Yamagata lineage), and BR/08 (Victoria lineage)] were obtained from the Influenza Division at the Centers for Disease Control and Prevention. Stocks of viruses were grown in the allantoic cavities of 10-day-old embryonated chicken eggs for 72 h at 33 °C, and aliquots were stored at −80 °C until use. Virus titers were determined by calculating the 50% tissue culture infectious dose (TCID_50_/mL)^68^, using Madin-Darby canine kidney (MDCK) cells obtained from the American Type Culture Collection (Manassas, VA).

### NAI susceptibility

The NAI oseltamivir carboxylate (oseltamivir), zanamivir, and peramivir were dissolved in distilled water and filter-sterilized, and stocks were stored at −20 °C until use. Susceptibility to NAIs was assessed in a fluorescence-based assay using 100 μM fluorogenic substrate 2′-(4-methylumbelliferyl)-α-D-N-acetylneuraminic acid (MUNANA) (Sigma–Aldrich, St. Louis, MO)^69^. The concentration of drug required to inhibit a standardized amount of NA activity by 50% (IC_50_) was calculated using GraphPad Prism 5 software (GraphPad Software, La Jolla, CA).

### Assessment of influenza B virus pathogenicity in different mouse strains

Female 6-week-old immunocompetent BALB/c and immunocompromised NOD *scid* and BALB *scid* mice (The Jackson Laboratory, Bar Harbor, ME) were lightly anesthetized with isoflurane and inoculated intranasally with 30 µL of 10^4^ or 10^5^ TCID_50_ of influenza B viruses (*n* = 5/group). Animals were monitored daily for signs of disease (activity level, ruffled fur, hunched-over posture) and body weight changes for 21 to 30 dpi. Mice that became severely hunched and lost more than 30% of their initial body weight were euthanized. To examine the virus load and the duration of virus replication in the respiratory tract, the BALF, nasal turbinates, and lung tissue samples from immunocompetent and immunocompromised mice inoculated with 10^4^ TCID_50_/30 µL of each virus were harvested at 3, 9, 13, 16, and 30 dpi (*n* = 3/group/time-point) and used for virus titration in MDCK cells^69^. To determine if any of the viruses exhibited extrapulmonary replication, virus detection was performed in brain, liver, spleen, and kidney samples collected from mice at 9 dpi.

### Differential leukocyte counts and flow cytometry

Inflammatory leukocytes (neutrophils, monocytes and total lymphocytes) in BALF were processed and analyzed by differential leukocyte counts by using a FORCYTE hematology analyzer (Oxford Science, Oxford, CT). To collect cells present in BALF, mice (*n* = 3/group) were euthanized and their lungs were flushed three times with 0.5 mL of PBS supplemented with 2 mM EDTA via an 18-gauge needle catheter inserted through the trachea. BALF samples were centrifuged at 2,000 rpm for 10 min, and neat pellets were resuspended in cold PBS and subjected to automated cell counting.

Analyzer generates cell count differential by constructing a distribution cytogram based on the relative size (impedance) and complexity (light scatter) of cells in the sample. BALB/c, NOD *scid*, and BALB *scid* mice (n = 3/group) were inoculated with sterile 1 × PBS, sacrificed at 9 dpi and were used to determine induction levels in control animals (referred to as the limit of detection).

For flow cytometry analysis of the immune cells present in BALF (NK, B, CD4^+^ T, and CD8^+^ T-cells), cells were blocked with mouse BD Fc block (BD Biosciences, Franklin Lakes, NJ) in staining medium ( 0.5% FBS in PBS) for 10 min at 4 °C then stained with CD3-APC, CD4-FITC, CD8-PE, CD19-PE-Cy7, and CD49b-APC-Cy7 antibodies (BD Biosciences) for 30 min at 4 °C. Cellular populations were gated (NK: CD3^−^ CD49b^+^; B: CD3^−^ CD19^+^; CD4^+^ T: CD3^+^ CD4^+^, and; CD8^+^ T-cells: CD3^+^ CD8^+^) and quantified by using FlowJo software version 7.6.1.

### Cytokine and chemokine analysis in lung tissue homogenates

At 3 and 9 dpi, the concentrations of each of 25 cytokines/chemokines were measured in the lung homogenates (*n* = 5 to 6/group) by using a MYCTOMAG-70K-PMX MILLIPLEX® MAP mouse 25-plex cytokine/chemokine panel (EMD Millipore, Billerica, MA) in accordance with the manufacturer’s instructions. For each cytokine/chemokine, the standard curve ranged from 3.2 to 10,000 pg/mL. The multiplex plates were read on a Luminex 100/200 analyzer, using the xPonent data acquisition and analysis software.

### Serologic assay

Blood samples were collected by retro-orbital bleed from surviving mice at 30 dpi. Serum from whole-blood samples was separated and harvested by centrifugation at 5,000 rpm for 10 min and then stored at −20 °C until use. Sera were treated with receptor-destroying enzyme (Denka Seiken Co., Ltd., Tokyo, Japan) for 16 to 18 h at 37 °C then heat-inactivated at 56 °C for 30 min. The hemagglutination inhibition (HI) titers were determined in serially diluted sera by using packed 0.5% turkey red blood cells.

### Lung immunohistopathologic and immunohistochemical (IHC) evaluation

The lungs (*n* = 4/group) were inflated *in situ* via tracheal infusion with 10% neutral-buffered formalin solution (vol/vol; NBF, ThermoFisher Scientific, Waltham, MA). The lungs were then removed and fixed in NBF for at least 2 weeks before being embedded and sectioned. One slide containing sections of all the lung lobes of each mouse was used for the evaluations. Tissue sections were stained with hematoxylin and eosin (HE) or subjected to immunohistochemical (IHC) staining with polyclonal goat antiserum raised against the HA glycoprotein of B/Florida/04/2006 (Yamagata lineage) virus. Additional podoplanin (gp38) (eBioscience, San Diego, CA) and surfactant C (Santa Cruz Biotechnology, Santa Cruz, CA) staining were performed for blinded pathology evaluation. The extent of virus spread and pulmonary lesions were quantified by first capturing digital images of whole-lung sections stained for viral antigen by using an Aperio ScanScope XT Slide Scanner (Aperio Technologies, Vista, CA) then manually outlining fields with areas containing viral antigen. The percentage of each lung field with active virus infection was calculated using the Aperio ImageScope software. HE-stained histologic sections were evaluated for 5 types of pulmonary lesions which were individually graded on a scale from 0 to 5 as follows: 0 = no lesions; 1 = minimal, focal to multifocal, inconspicuous; 2 = mild, multifocal, concpicuous; 3 = moderate, multifocal, prominent; 4 = marked, multifocal or coalescing, lobar; 5 = severe, extensive, and diffuse, often with multilobar consolidation. The severity grades were converted to semiquantitative scores as follows: 0 = 0; 1 = 2; 1.5 = 8; 2 = 15; 2.5 = 25; 3 = 40; 3.5 = 60; 4 = 80; 4.5 = 90, and; 5 = 100.

### Drug efficacy in immunocompetent and immunocompromised mice

BALB/c and BALB *scid* mice were lightly anesthetized by inhalation of isoflurane and were inoculated intranasally with a predetermined 5 × 50% mouse lethal dose (5 MLD_50_, equivalent to 2.5 × 10^5^ TCID_50_) of BR/08 in 30 µL of PBS. Peramivir (at 75 mg/kg/day in 0.1 mL) was administered to BALB/c or BALB *scid* mice (*n* = 5/group) by IM injection once a day in a single (1×: +24 hpi), double (2×: +24 and +72 hpi) or quadruple (4×: +24, +72, +120, and +168 hpi) dose. Control animals received IM injection of sterile RNAse-free water (4×: 24, 72, 120, and 168 hpi). The mice were observed daily for clinical signs, weight loss, and survival. Additionally, separate groups of control mice and mice treated with a 1× dose or 2× dose were euthanized at 4, 6, 9, 13, 16, and 30 dpi (*n* = 3/group/time-point), and their nasal turbinates, lungs, and BALF were collected in order to determine the virus titers by TCID_50_ assay in MDCK cells.

### Emergence of drug-resistant variants

At 9, 16, and 30 dpi, lung tissue homogenates from BALB/c and BALB *scid* mice (*n* = 3/group/time-point), inoculated with BR/08 virus and treated with peramivir were subjected for plaque assay. Briefly, monolayers of confluent MDCK cells were infected with the 10-fold dilution of lung tissue homogenates. After 1 h of incubation at 35 °C, the cells were washed with sterile PBS and overlaid with a 0.9% immunodiffused agarose-medium mixture with 1 μg/mL of L-1-tosylamide-2-phenylmethyl chloromethyl ketone (TPCK)-treated trypsin. At 96 hpi, 15 single-plaque colonies were picked and resuspended in medium. The RNeasy Kit (Qiagen, Chatsworth, CA) was used to extract viral RNA, and the One Step RT-PCR kit (Qiagen, Chatsworth, CA) was used to amplify NA gene. Universal primers were used to amplify the full-length NA gene^70^. The sequences were determined by the Hartwell Center for Bioinformatics and Biotechnology at St. Jude Children’s Research Hospital by using the BigDye1 Terminator v3.1 Cycle Sequencing Kit and were analyzed on an Applied Biosystems 3730xl DNA sequencer (Applied Biosystems, ThermoFisher Scientific, Waltham, MA). DNA sequences were completed and edited using the Lasergene sequence analysis software package (DNASTAR, Madison, WI).

### Statistical analysis

Virus titers, cell counts, and cytokine/chemokines levels were compared by one-way analysis of variance (ANOVA) with Bonferroni’s multiple comparison post-test (GraphPad Prism 5.0 software). The Kaplan-Meier method was used to estimate the probability of survival in mice inoculated with BR/08, and the log-rank test was used to compare survival rates in the control and treatment groups.

## Electronic supplementary material

Supplementary Data
