# Supplementary material for: Pathogenicity and peramivir efficacy in immunocompromised murine models of influenza B virus infection
Source: Sci Rep. 2017 Aug 4;7:7345. doi: 10.1038/s41598-017-07433-z (PMC5544712; doi:10.1038/s41598-017-07433-z)
Supplement: Supplementary file 1 — Supplementary Data [file 41598_2017_7433_MOESM1_ESM.pdf]

Pathogenicity and peramivir efficacy in immunocompromised murine models of influenza B virus infection

Authors: Philippe Noriel Q. Pascua[1], Heba H. Mostafa[1], Bindumadhav M. Marathe[1], Peter Vogel[2], Charles J. Russell[1], Richard J. Webby[1], Elena A. Govorkova[1#]

Affiliations

[1]Department of Infectious Diseases, St. Jude Children's Research Hospital, Memphis, Tennessee, USA; [2]Veterinary Pathology Core, St. Jude Children's Research Hospital, Memphis, Tennessee, USA

Running title: Immunocompromised murine models of influenza B virus infection

#To whom correspondence should be addressed: Elena A. Govorkova, Department of Infectious Diseases, St. Jude Children's Research Hospital, 262 Danny Thomas Place, Memphis, TN 38105-3678. Phone: (901) 595-2243. Fax: (901) 595-8559. Email: elena.govorkova@stjude.org

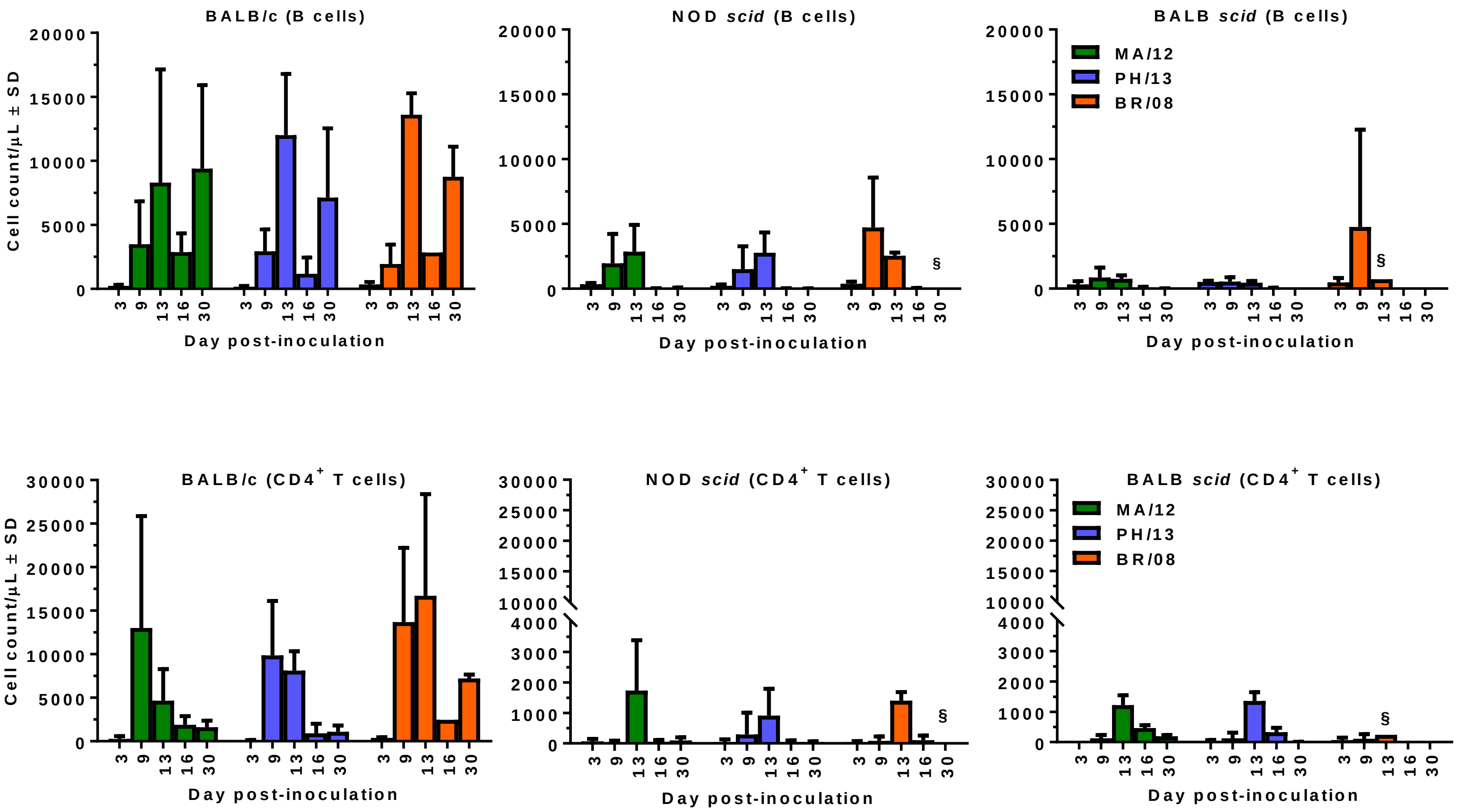

Supplementary Figure 1. Induction of immune cells in the BALF of immunocompetent and immunocompromised mice inoculated with influenza B viruses. BALB/c, NOD *scid*, and BALB *scid* mice were inoculated with viruses as described in the legend for Figure 2. Lymphocyte cell counts were determined in the BALF of inoculated mice at 3, 9, 13, 16, and 30 dpi by flow cytometry. Bars represent mean values ± SD (*n* = 3/group/time-point). [§]Detection in one mouse only.

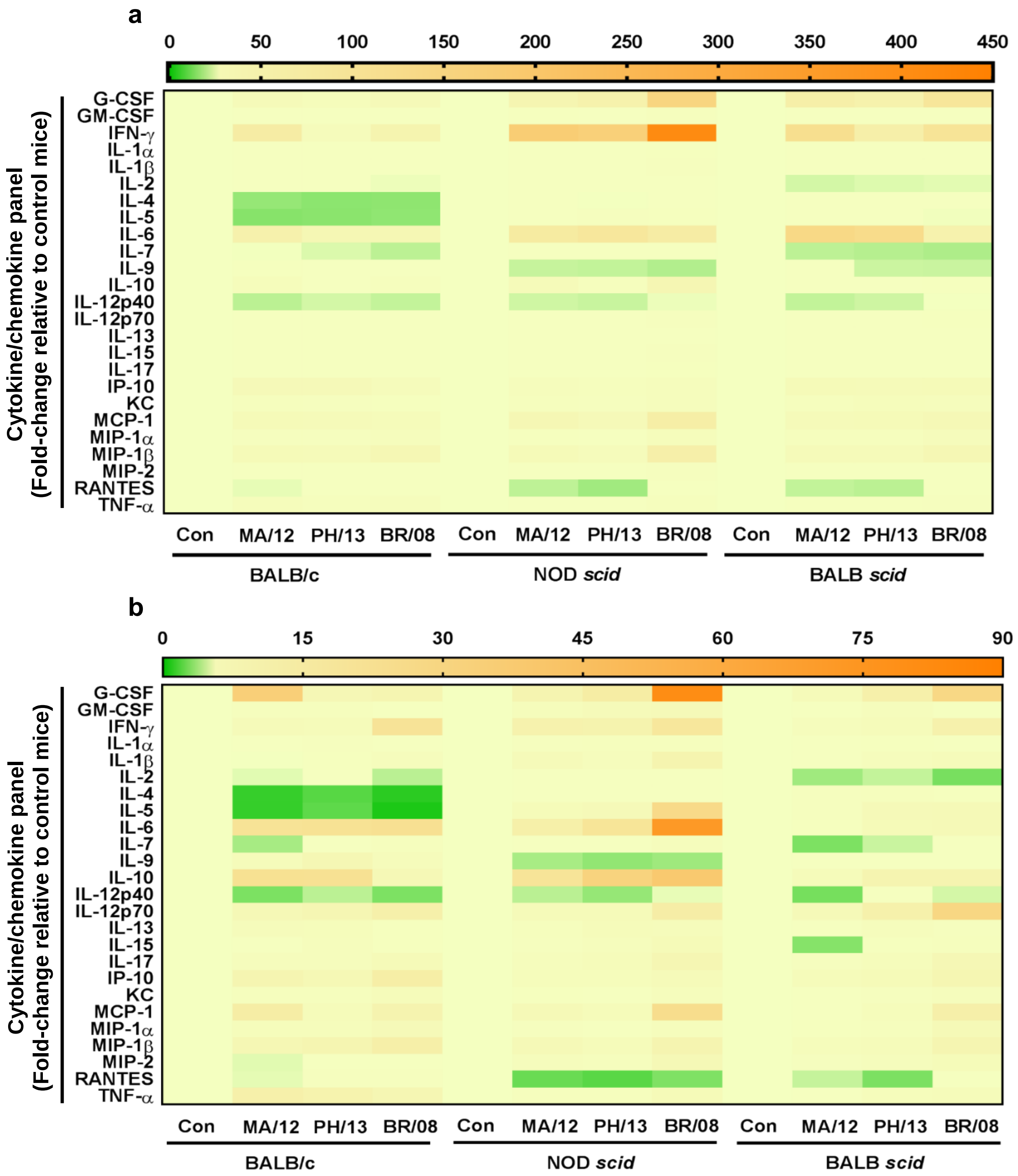

Supplementary Figure 2. Pattern of cytokine and chemokine induction in the lungs of immunocompetent and immunocompromised mice inoculated with influenza B viruses. BALB/c, NOD *scid*, and BALB *scid* mice were inoculated with viruses as described in the legend for Figure 2. The patterns of cytokine and chemokine production in the lungs of mice inoculated with influenza B viruses are shown. The expression levels of 25 cytokines and

chemokines were assayed in lung homogenates (*n* = 5–6/group) at 3 and 9 dpi. The level of each cytokine/chemokine tested was normalized to that of control (PBS-inoculated) animals (1, shown in yellow). The fold changes in the concentration of each cytokine or chemokine ranged from -1 (green) to 450 (a) or 90 (b) (orange).
